# Periprosthetic Fracture through a Unicortical Tracking Pin Site after Computer Navigated Total Knee Replacement

**DOI:** 10.1155/2018/2381406

**Published:** 2018-09-16

**Authors:** Matt Blue, Christian Douthit, Joel Dennison, Cyrus Caroom, Mark Jenkins

**Affiliations:** Department of Orthopaedic Surgery and Rehabilitation, Texas Tech University Health Sciences Center, 3601 4th St. MS 9436, Lubbock, TX 79436, USA

## Abstract

A rare complication from computer-navigated total knee arthroplasty is a fracture through the insertion site of a tracking pin. These pins are inserted across the femoral and tibial shafts either bicortically, transcortically, or unicortically and have a reported fracture incidence of 1.38%, with all published cases occurring after bicortical pin placement. In this case, a 60-year-old female suffered a femoral shaft fracture through a unicortically inserted computer navigation tracking pin 6 weeks after total knee arthroplasty. Her fracture was successfully fixated with an intramedullary nail with retention of the knee prosthesis. This case is important as it records the risk for a postoperative fracture through a unicortically inserted computer navigation pin.

## 1. Introduction

We present a case of a femoral fracture through a previous tracking pin site after computer-navigated total knee arthroplasty (TKA). The usage of computer-navigated TKA has gained popularity due to the benefit of more accurate femoral and tibial cuts, but this technique comes with unique adverse event possibilities. One of the possible adverse events is a periprosthetic fracture through the insertion sites of pins used to mount the robotic tracking system for extremity mapping [1]. All previously reported cases of femoral fractures of this nature have been seen in bicortically placed tracking pins [1–3]. In our case, we present a patient with a fracture through a computer-navigated total knee arthroplasty tracking pin site placed unicortically. To our knowledge, a fracture through a unicortical pin site has not been previously reported for this procedure.

## 2. Case

A 60-year-old female with osteoarthritis of the right knee underwent Mako/Stryker robotic-assisted total knee arthroplasty. The patient's past medical history consisted of hypertension. No medical conditions predisposing the patient to a fracture were noted. A midvastus approach was used, and trackers were placed with unicortically inserted pins in the tibial shaft and femoral shaft for the robotic tracking system. The operation was performed without complication, and the tracking pins were removed manually at the conclusion of the case. Postoperative radiology reports noted proper positioning of prosthesis with no acute fracture.

Physical therapy was begun on postoperative day 1 with partial weight bearing with a front wheel walker with PT assistance and was able to walk the entire hospital corridor two times. Patient was discharged on postoperative day 2 with outpatient physical therapy arrangements. Patient had a satisfactory postoperative course.

**Figure 1:**
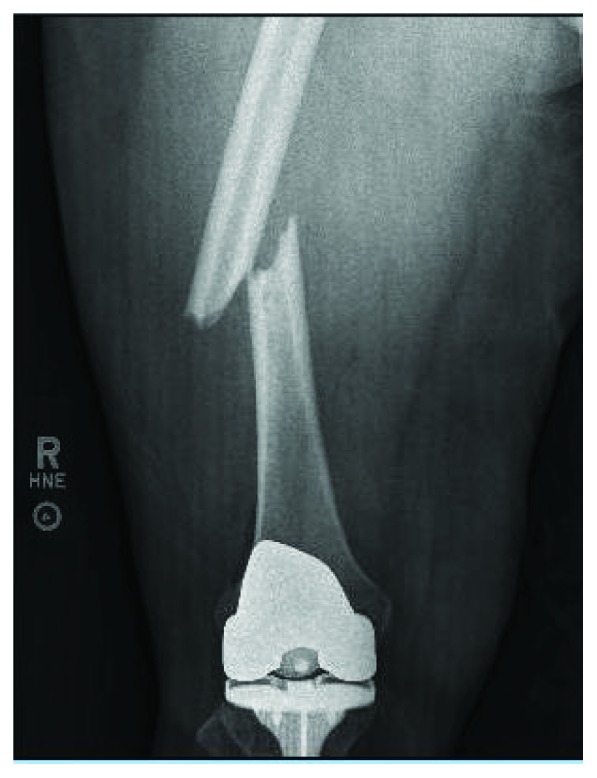
Anteroposterior radiograph of the right femur demonstrating a fracture through femoral diaphysis.

**Figure 2:**
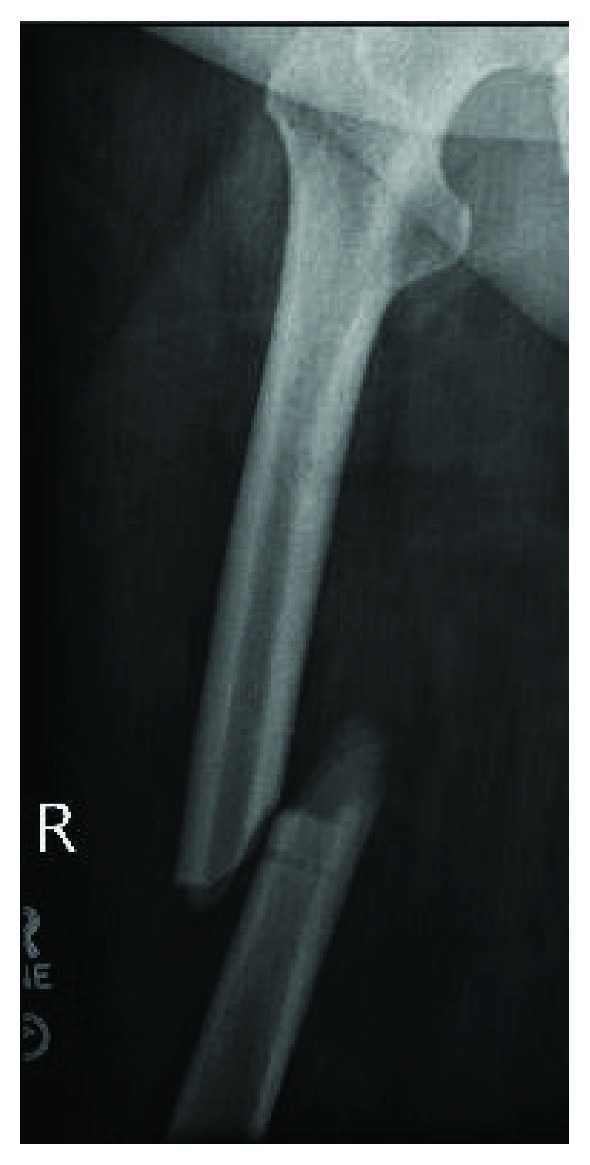
Right femur radiograph with a unicortical tracking pin site clearly visible.

**Figure 3:**
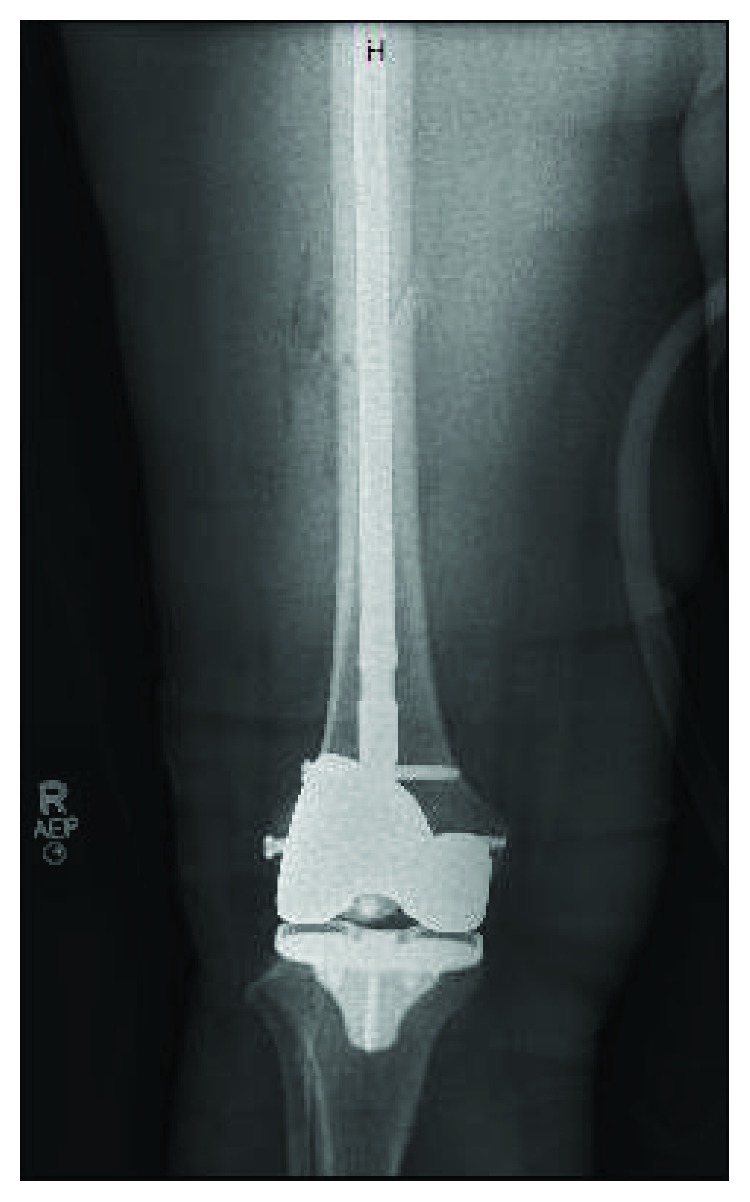
Anteroposterior radiograph of the right femur after intramedullary nailing of the femoral shaft fracture.

**Figure 4:**
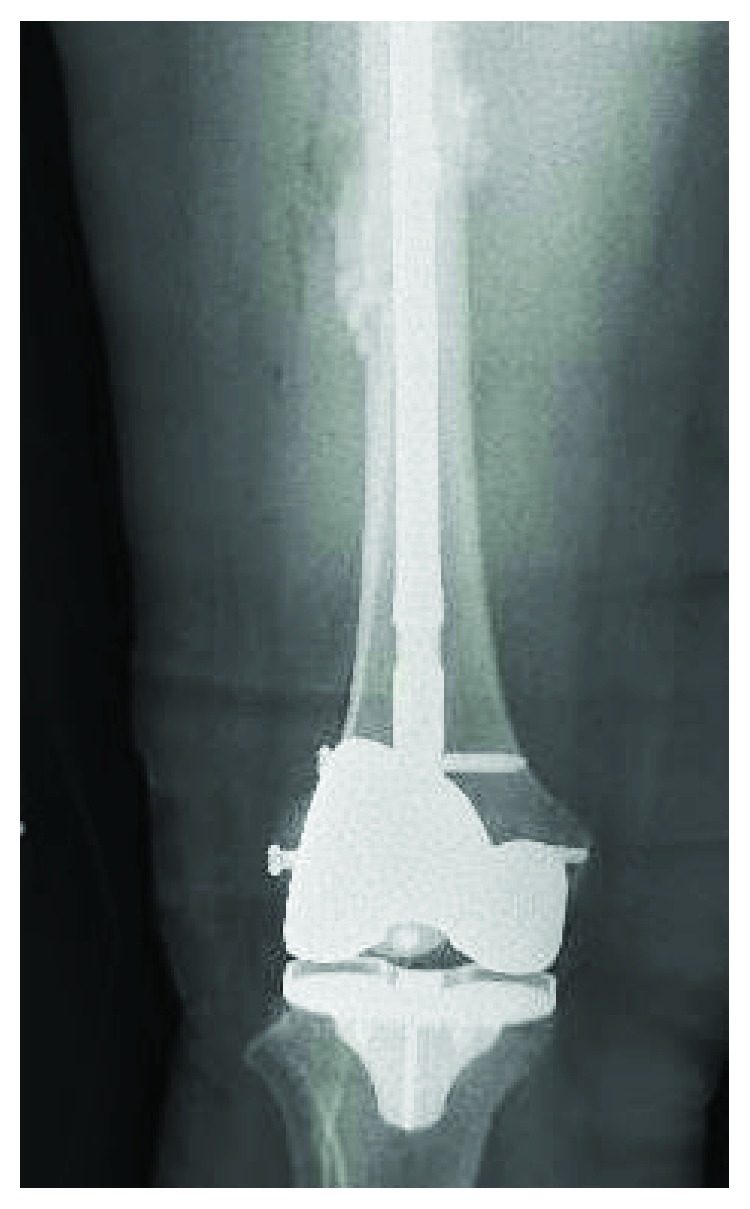
Anteroposterior radiograph of right femur with a healed fracture and stable orthopedic hardware.

The patient had an uncomplicated recovery until 6 weeks postoperatively when she was at work and sustained a ground level fall due to acute pain of the right thigh and her leg “giving out.” Patient was unable to bear weight on the extremity. She presented to the emergency department where she was found to have a complete, oblique fracture through the midshaft of the right femur (Figure 1). On radiography, the femoral tracking pin site is clearly visible at the location of the fracture in the distal segment (Figure 2). The patient was taken to the OR and underwent intramedullary nailing of the right femur with retention of right knee prosthesis hardware (Figure 3). CT scan of right lower extremity confirmed evidence of a fracture through the prior surgery pin tracks. Following intramedullary nail placement, the patient had a normal postoperative course. Final films at 6 months showed a healed fracture with stable orthopedic hardware (Figure 4). At this time, the patient was pain free, ambulating without issue, and elected to proceed with an as needed follow-up.

## 3. Discussion

Any abrupt change in contour or consistency of bone, such as the insertion of external fixator pins or computer-assisted navigation pins, can act as a weak point within the bone where stress is concentrated. Femur fractures through these pin sites have been documented in pediatrics for external fixator pins and in adults in bicortically placed computer-assisted navigation pins in total knee arthroplasties [1–3].

The usage of computer navigated TKA has the benefit of more accurate femoral and tibial cuts but requires the insertion of threaded guidance pins in the femur and tibia that are removed postoperatively. These tracking pins can be placed either bicortically or transcortically through the femur, and removal of these pins leaves behind a bone defect that has been documented as a potential site of stress and nidus for a fracture [1]. While the complication of a femoral fracture through a navigation pin site is rare, it is serious as it requires readmission to the hospital and additional operations, which both have significant associated cost and morbidity. A review of pin site complications performed by Thomas et al. documents a fracture incidence through femoral pin tracker sites of 1.38% in a series of 385 TKAs [2]. In this case series, as well as in other reported cases, all fractures seen through previous pin sites have been through bicortically inserted tracking pins [1–5]. A review of 984 computer-navigated TKAs with all femoral tracking pins placed unicortically performed by Owens and Swank showed no fractures occurring when the unicortical pin placement technique was used [6]. In our case, we present a patient with a fracture through a unicortical tracking pin site which, to our knowledge, has not been previously reported for this procedure.

This case illustrates that the risk of a fracture through a prior pin site is present for both unicortical and bicortical pins. While the risk of a fracture at these sites has been reported previously, all documented cases were seen in bicortical pins, and it is important to realize that the risk exists regardless of the pinning technique utilized.

## References

[1] Beldame J., Boisrenoult P., Beaufils P. (2010). Pin track induced fractures around computer-assisted TKA. *Orthopaedics & Traumatology: Surgery & Research*.

[2] Thomas A., Pemmaraju G., Nagra G., Bassett J., Deshpande S. (2015). Complications resulting from tracker pin-sites in computer navigated knee replacement surgery. *Acta Orthopaedica Belgica*.

[3] Wysocki R. W., Sheinkop M. B., Virkus W. W., Della Valle C. J. (2008). Femoral fracture through a previous pin site after computer-assisted total knee arthroplasty. *The Journal of Arthroplasty*.

[4] Jung K. A., Lee S. C., Ahn N. K., Song M. B., Nam C. H., Shon O. J. (2011). Delayed femoral fracture through a tracker pin site after navigated total knee arthroplasty. *The Journal of Arthroplasty*.

[5] Li C. H., Chen T. H., Su Y. P., Shao P. C., Lee K. S., Chen W. M. (2008). Periprosthetic femoral supracondylar fracture after total knee arthroplasty with navigation system. *The Journal of Arthroplasty*.

[6] Owens R. F., Swank M. L. (2010). Low incidence of postoperative complications due to pin placement in computer-navigated total knee arthroplasty. *The Journal of Arthroplasty*.

